# Exploring substitution random functions composed of stationary multi-Gaussian processes

**DOI:** 10.1007/s00477-024-02662-x

**Published:** 2024-02-09

**Authors:** Julien Straubhaar, Philippe Renard

**Affiliations:** The Centre for Hydrogeology and Geothermics (CHYN), University of Neuchâtel, Emile-Argand 11, 2000 Neuchâtel, Switzerland

**Keywords:** Stochastic simulation, Composition of Gaussian processes, Connectivity properties, Conditioning

## Abstract

Simulation of random fields is widely used in Earth sciences for modeling and uncertainty quantification. The spatial features of these fields may have a strong impact on the forecasts made using these fields. For instance, in flow and transport problems the connectivity of the permeability fields is a crucial aspect. Multi-Gaussian random fields are the most common tools to analyze and model continuous fields. Their spatial correlation structure is described by a covariance or variogram model. However, these types of spatial models are unable to represent highly or poorly connected structures even if a broad range of covariance models can be employed. With this type of model, the regions with values close to the mean are always well connected whereas the regions of low or high values are isolated. Substitution random functions (SRFs) belong to another broad class of random functions that are more flexible. SRFs are constructed by composing ($Z=Y\circ T$) two stochastic processes: the directing function *T* (latent field) and the coding process *Y* (modifying the latent field in a stochastic manner). In this paper, we study the properties of SRFs obtained by combining stationary multi-Gaussian random fields for both *T* and *Y* with bounded variograms. The resulting SRFs *Z* are stationary, but as *T* has a finite variance *Z* is not ergodic for the mean and the covariance. This means that single realizations behave differently from each other. We propose a simple technique to control which values (low, intermediate, or high) are connected. It consists of adding a control point on the process *Y* to guide every single realization. The conditioning to local values is obtained using a Gibbs sampler.

## Introduction

Random fields play a key role in Earth sciences (Chilès and Delfiner 1999; Lantuéjoul 2002). Indeed, stochastic spatial (or temporal) simulation is one of the most important tools for uncertainty quantification allowing the forecasting of natural phenomena and risk assessment. For example, the modeling of groundwater flow and solute transport underground requires hydraulic conductivity fields as input for the numerical code solving the flow equations. Generating an ensemble of stochastic hydraulic conductivity fields is a key step for representing and propagating the uncertainty in this system. In general, the goal of geostatistical simulation techniques is to provide methods to generate random fields that respect some spatial features and honor conditioning data if present. In particular, the size, shape, orientation, and connectivity are spatial characteristics that need to be controlled by a simulation technique to represent realistic geological structures.

Non-parametric methods such as multiple-point statistics (Mariethoz and Caers 2014), or machine learning techniques such as generative adversarial networks (Goodfellow et al. 2016) allow generating complex realistic random fields, providing that a training data set is available. Such algorithms are extremely flexible because they do not require inferring the parameters of an analytical statistical model, but they can be difficult to set up (parameters, neural network architecture) and can be time-consuming (learning structures from training data).

Conversely, simulation methods based on analytical models are easier to set up, and faster, but they are limited in terms of the complexity of the generated structures. A broad description of such algorithms can be found in Chilès and Delfiner (1999). The most standard simulation techniques are based on the multi-Gaussian random field (or function) (GRF) model (see Chilès and Delfiner 1999, p. 394–395) also known as Gaussian processes (Rasmussen and Williams 2006). These random functions assume a multivariate Gaussian distribution as their spatial statistical law and can be defined by a covariance model, describing the statistics between any pair of points according to their relative location. Considering stationary GRFs, anisotropies, and orientations are easy to handle with the covariance model, but there is no mean to control and simulate various connectivity patterns (Renard and Allard 2013): values around the mean are always well connected (in more than one dimension), and low- or high-value regions are isolated.

But, connectivity patterns in hydraulic conductivity fields have a very strong impact on groundwater flow and solute transport (Zinn and Harvey 2003; Knudby and Carrera 2005; Renard and Allard 2013; Tyukhova and Willmann 2016): low-conductivity connected regions can act as a barrier to the flow, whereas high-conductivity connected regions enable flow paths and fast mass transfer. Zinn and Harvey (2003) proposed a simple technique to get low or high values connected from a GRF. A zero mean GRF is transformed by taking the absolute value which produces a low-value connected region, or high-value connected by reversing the sign. Then a normal-score transform is applied to ensure that the marginal distribution is Gaussian and finally, a coordinate rescaling allows adjusting the covariance. However, this strategy produces peaks (non-derivable) for extreme value areas, and honoring the conditioning data is difficult.

In this article, we propose to modify stochastically GRFs to enrich the range of connectivity patterns that can be simulated while keeping the ability of conditioning. We use the framework of substitution random function (SRF), defined as the composition of two independent random processes, $Z(x)=Y(T(x))$. This family of random functions was introduced by Lantuéjoul (1993). In his book, Lantuéjoul (2002) derives properties on *Z* assuming that the directing function (latent field *T*) has stationary increments and that the coding function is stationary. Moreover, he describes an algorithm for conditional simulation and proposes examples for categorical fields. These examples are based on Chentsov simulation as the directing function and a Markov chain as the coding process.

To our knowledge, only a few applications of SRF can be found in the scientific literature. Recently, Allard et al. (2020) developed a simulation technique for generating space-time random fields, where the coding process consists of a cosine function with a random amplitude and a random phase. Emery (2008) develops SRF methods for continuous simulation, based on a multivariate directing function composed of independent latent GRFs with unbounded variograms, and on a GRF as the coding process with separable covariance. In this way, a finite integral range can be obtained for the resulting SRF and, as a consequence, its ergodicity. Illustrations on a pollution data set show the ability of SRF to generate realizations of pollutant concentration depicting clustering of high values with more spatial contrasts than classical GRF.

In this work, another point of view is adopted, we investigate how to modify a stationary GRF considered as a latent field (directing function) with the use of a continuous coding process defined as a uni-dimensional multi-Gaussian process, to obtain various connectivity patterns. Considering that the directing function has a bounded variogram (finite variance) implies that the resulting SRF is not ergodic for the mean and the covariance. Different characteristics will be depicted from one realization to another. We introduce a *control point* on the coding process to guide the simulation towards the desired connectivity property. The idea is to condition the coding process at the mean of the latent field. Moreover, we derive an expression for the expectation of the probability distribution function of the simulated values in a single realization for this case. This allows applying an anamorphosis (normal score transform, or more generally change of distribution) while preserving the ability to generate conditional simulations.

The paper is organized as follows. Theoretical developments are proposed in Sect. 2 to 4, illustrations are presented in Sect. 5, and finally, a discussion and conclusions are given in Sect. 6.

## Substitution random functions (SRF) as a composition of multi-Gaussian processes

A substitution random function (SRF) *Z* on ${\mathbb {R}}^d$ with values in ${\mathbb {R}}$ is a composition

1 $$\begin{aligned} Z(x) = Y(T(x)), x\in {\mathbb {R}}^d, \end{aligned}$$

where *T* and *Y*, respectively called the directing function and the coding process, are two independent random functions. The directing function is assumed to have stationary increments, i.e. the distribution of $T(x)-T(x+h)$ depends only on the lag vector *h*, and the coding process is assumed to be stationary, i.e. the distribution of *Y* at a family of locations $\{t_i\}$ is the same as the distribution at $\{t_i+t\}$ for any *t*. Note that the directing function can be multivariate, *T* with values in ${\mathbb {R}}^k$, which implies a coding process on ${\mathbb {R}}^k$. In the following, we consider the univariate case ($k=1$). Under these assumptions, some properties on *Z* are known. In particular, the SRF *Z* is stationary with same distribution at any point as *Y* (Lantuéjoul 2002), i.e. denoting ${\mathcal {D}}_V$ the point distribution of a random function *V*,

2 $$\begin{aligned} {\mathcal {D}}_Z = {\mathcal {D}}_Y. \end{aligned}$$

Moreover, denoting $C_Y(s) = {\text {Cov}}(Y(t), Y(t+s))$ the covariance function of *Y* (assuming it exists), the covariance function of *Z*, $C_Z(h) = {\text {Cov}}(Z(x), Z(x+h))$, is expressed as (Lantuéjoul 2002)

3 $$\begin{aligned} C_Z(h) = {\mathbb {E}}\left[ C_Y(T(x+h)-T(x))\right] . \end{aligned}$$

In the following, we focus on the case where

- *T* is a stationary GRF on ${\mathbb {R}}^d$, defined by its mean $\mu _T$ and covariance function $C_T$,
- *Y* is a stationary GRF on ${\mathbb {R}}$, defined by its mean $\mu _Y$ and covariance function $C_Y$.

In this framework, $T(x+h) - T(x)$ follows a zero-mean normal distribution of variance $2(C_T(0)-C_T(h))$, and the covariance of *Z* (Eq. (3)) can be written as

4 $$\begin{aligned} C_Z(h) = {\mathbb {E}}_{t\sim {\mathcal {N}}(0, 2(C_T(0)-C_T(h)))}\left[ C_Y(t)\right] . \end{aligned}$$

To facilitate the notations, we introduce

5 $$\begin{aligned} g_Y(s^2)= & {} {\mathbb {E}}_{t\sim {\mathcal {N}}(0, s^2)}\left[ C_Y(t)\right] \nonumber \\= & {} \frac{1}{\sqrt{2 \pi } s} \int _{-\infty }^{\infty } C_Y(t) \exp \left( - \frac{1}{2} \frac{t^2}{s^2} \right) dt \end{aligned}$$

which is the mean of the covariance function of *Y* according to the centered normal distribution of variance $s^2$, with the convention that $g_Y(0) = C_Y(0)=\sigma _Y^2$. With this notation we get

6 $$\begin{aligned} C_Z(h) = g_Y\left[ 2(C_T(0)-C_T(h))\right] . \end{aligned}$$

### Integral range and ergodicity

For a stationary random function *V* on ${\mathbb {R}}^d$, the average value over a domain $\Omega \subset {\mathbb {R}}^d$,

7 $$\begin{aligned} V(\Omega ) = \frac{1}{\vert \Omega \vert }\int _\Omega V(x) dx, \end{aligned}$$

is an unbiased estimator of the point mean $\mu _V = {\mathbb {E}}(V(x))$ (independent of *x*), since ${\mathbb {E}}[V(\Omega )]=\mu _V$. If its variance tends to zero when $\Omega$ grows to ${\mathbb {R}}^d$ (a property known as ergodicity for the mean, see Lantuéjoul (2002)), then this means that $\mu _V$ can be estimated by taking the average value of a single realization over a large domain. Assuming *V* second-order stationary with covariance $C_V$, the variance of $V(\Omega )$ is expressed as

8 $$\begin{aligned} {\text {Var}}[V(\Omega )]= & {} \frac{1}{\vert \Omega \vert ^2}\int _\Omega \int _\Omega {\text {Cov}}(V(x), V(y)) dx dy \nonumber \\{} & {} = \frac{1}{\vert \Omega \vert ^2}\int _\Omega \int _\Omega C_V(x-y) dx dy. \end{aligned}$$

It is linked to the integral range of *V* defined as

9 $$\begin{aligned} I_R(V) = \lim _{\Omega \rightarrow {\mathbb {R}}^d} \vert \Omega \vert \frac{{\text {Var}}[V(\Omega )]}{C_V(0)}, \end{aligned}$$

that can be computed, if $C_V$ is integrable, as (Lantuéjoul 2002)

10 $$\begin{aligned} I_R(V) = \frac{1}{C_V(0)}\int _{{\mathbb {R}}^d} C_V(h) dh. \end{aligned}$$

This gives a simpler expression for the variance of $V(\Omega )$ for a large domain $\Omega$,

11 $$\begin{aligned} {\text {Var}}[V(\Omega )] \approx \frac{C_V(0)\cdot I_R(V)}{\vert \Omega \vert } = \frac{1}{\vert \Omega \vert }\int _{{\mathbb {R}}^d} C_V(h) dh. \end{aligned}$$

Assuming that the covariance function $C_T$ of the GRF *T* decreases towards 0 (when $\vert h\vert$ increases), such that the integral range of *T*, $I_R(T)$ (Eq. (10)), is finite, implies $\text {Var(T}(\Omega ))\rightarrow \text {0}$ when $\Omega \rightarrow {\mathbb {R}}^d$, i.e. *T* is ergodic for the mean.

However, even if the covariance function of *Y* is rapidly decreasing towards 0, from Eq. (6) the covariance function of *Z* is decreasing but lower bounded by $g_Y(2C_T(0))=g_Y(2\sigma _T^2)$, which is strictly positive since the variance of *T* is finite. This implies by Eq. (10) that $I_R(Z)=+\infty$, and by Eq. (8) that ${\text {Var}}(Z(\Omega ))\geqslant g_Y(2\sigma _T^2)>0$. More precisely, if we assume that $C_Z(h) - g_Y(2\sigma _T^2)$ is sufficiently rapidly decreasing such that $A=\int _{{\mathbb {R}}^d} \left( C_Z(h) - g_Y(2\sigma _T^2)\right) dh$ is finite, then, with ${\varvec{1}}_\Omega$ the indicator function of the domain $\Omega$,

12 $$\begin{aligned} 0&\leqslant {\text {Var}}[Z(\Omega )] - g_Y(2\sigma _T^2) = \frac{1}{\vert \Omega \vert ^2}\int _\Omega \int _\Omega \left( C_Z(x-y)\right. \nonumber \\&\quad \left. -g_Y(2\sigma _T^2)\right) dx dy \nonumber \\&= \frac{1}{\vert \Omega \vert ^2}\int _\Omega \left[ \int _{{\mathbb {R}}^d} \left( C_Z(h)-g_Y(2\sigma _T^2)\right) \cdot \varvec{1}_\Omega (y+h) dh\right] dy \nonumber \\&\leqslant \frac{1}{\vert \Omega \vert ^2}\int _\Omega A dy \leqslant \frac{A}{\vert \Omega \vert } \underset{\Omega \rightarrow {\mathbb {R}}^d}{\longrightarrow } 0, \end{aligned}$$

and ${\text {Var}}[Z(\Omega )]$ converges to $g_Y(2\sigma _T^2)$ when $\Omega \rightarrow {\mathbb {R}}^d$, i.e., for a large domain $\Omega$,

13 $$\begin{aligned} {\text {Var}}[Z(\Omega )]\approx g_Y(2\sigma _T^2)>0. \end{aligned}$$

As ${\text {Var}}[Z(\Omega )]$ does not vanish, *Z* is not ergodic for the mean. A similar analysis of the ergodicity of the covariance can show that it is not ergodic as well for the covariance. This will be illustrated graphically with some examples in Sect. 5.

### Ensemble covariance and distribution of SRF

The non-ergodicity of *Z* for the mean and the covariance implies that one cannot infer the properties of *Z* from a single realization. For example, to estimate its covariance function (Eq. (6)), an ensemble of realizations $\{Z_i\}_{i \in I}$ has to be considered on a large domain $\Omega$,

14 $$\begin{aligned} C_Z(h) \approx {\text {Cov}}_{(x, i)\in \{x\in \Omega :x+h\in \Omega \}\times I}\left( Z_i(x), Z_i(x+h)\right) \end{aligned}$$

(with $\Omega$ and *I* equipped with the uniform distribution). The covariance function $C_Z$ is then referred to as an *ensemble covariance function*. Similarly the cumulative distribution function (CDF) of *Z*, which is known to be the CDF of ${\mathcal {N}}(\mu _Y, \sigma _Y^2)$ (see Eq. (2)), can be estimated from the ensemble of realizations,

15 $$\begin{aligned} F_Z(z)= & {} \Phi \left( \frac{z-\mu _Y}{\sigma _Y}\right) \approx {\mathbb {E}}_{(x, i)\in \Omega \times I}\left[ \varvec{1}_{\leqslant z}(Z_i(x))\right] \nonumber \\{} & {} \approx {\mathbb {E}}_{i\in I}\left[ F_{Z_i}(z)\right] \end{aligned}$$

where $\varvec{1}_{\leqslant z}$ is the indicator function of the interval $]-\infty , z]$, and $\Phi$ the CDF of ${\mathcal {N}}(0, 1)$.

In the next sub-sections, we analyze the properties of single realizations. This is important since in practical applications single realizations are used as input for further computations.

### Covariance of single SRF realizations

To decouple the ensemble of realizations and the simulation domain in the estimation of the covariance (Eq. (14)), we use the fact that, for any random variables *U*, *V*, *W*,

16 $$\begin{aligned}{} & {} {\text {Cov}}(U,V)={\mathbb {E}}_{\sim W}\left[ {\text {Cov}}(U\,\vert \,W, V\,\vert \,W)\right] \nonumber \\{} & {} \quad + {\text {Cov}}_{\sim W}\left[ {\mathbb {E}}(U\,\vert \, W), {\mathbb {E}}(V\,\vert \, W)\right] . \end{aligned}$$

Hence,

17 $$\begin{aligned} C_Z(h)&\approx {\text {Cov}}_{(x, i)\in \{x\in \Omega :x+h\in \Omega \}\times I}\left( Z_i(x), Z_i(x+h)\right) \nonumber \\&= {\mathbb {E}}_{i}\left[ {\text {Cov}}_{x}(Z_i(x), Z_i(x+h))\right] + {\text {Cov}}_{i}\left[ {\mathbb {E}}_{x}(Z_i(x)), \right. \nonumber \\&\quad \left. {\mathbb {E}}_{x}(Z_i(x+h))\right] ,\nonumber \\&\approx {\mathbb {E}}_{i}\left[ C_{Z_i}(h)\right] +{\text {Var}}_i\left[ Z_i(\Omega )\right] . \end{aligned}$$

where $C_{Z_i}$ denotes the covariance of a single realization $Z_i$ computed over $\Omega$. Note that in the last step, *h* is assumed to be a small lag vector compared to the size of $\Omega$, such that in the second term, ${\mathbb {E}}_{x}(Z_i(x)) \approx {\mathbb {E}}_{x}(Z_i(x+h)) \approx Z_i(\Omega )$. Hence, the covariance function for a single realization, $C_{Z_i}$, is in mean equal to the ensemble covariance function $C_Z$, shifted by the variance of the average value over the simulation domain,

18 $$\begin{aligned} {\mathbb {E}}_{i}\left[ C_{Z_i}(h)\right] \approx C_Z(h) - {\text {Var}}[Z(\Omega )]. \end{aligned}$$

Writing $\overline{C}_{Z_{s}}(h) = {\mathbb {E}}_{i}\left[ C_{Z_i}(h)\right]$ the mean covariance function for single realization of *Z*, it follows by Eqs. (6), (13) and  (18) that, for a large domain $\Omega$,

19 $$\begin{aligned} \overline{C}_{Z_{s}}(h) \approx g_Y\left[ 2(\sigma _T^2-C_T(h))\right] - g_Y\left( 2\sigma _T^2\right) . \end{aligned}$$

Analytical expressions for $g_Y(s^2)$ are given in Table 1 in the case of classical covariance functions $C_Y$ of type Gaussian, exponential, and spherical, with a sill of $\sigma _Y^2$ and a range of $r_Y$. They are obtained by simple integrations (the result for the Gaussian and exponential models can also be found in Emery (2008)).

**Table 1:** Analytical expression of $g_Y(s^2)$ for classical covariance model $C_Y$ with sill $C_Y(0)=\sigma _Y^2$ and range $r_Y$; $\Phi$ is the CDF of ${\mathcal {N}}(0, 1)$

Model	$C_Y(t)$	$g_Y(s^2) ={\mathbb {E}}_{t\sim {\mathcal {N}}(0, s^2)}\left[ C_Y(t)\right]$
Gaussian	$\displaystyle \sigma _Y^2\exp \left( -3\frac{t^2}{r_Y^2}\right)$	$\displaystyle \sigma _Y^2\left( 1+6\frac{s^2}{r_Y^2}\right) ^{-1/2}$
Exponential	$\displaystyle \sigma _Y^2 \exp \left( -3\frac{\vert t\vert }{r_Y}\right)$	$\displaystyle 2 \sigma _Y^2\exp \left( \frac{9s^2}{2r_Y^2}\right) \left[ 1-\Phi \left( \frac{3s}{r_Y}\right) \right]$
Spherical	$\displaystyle \sigma _Y^2 \left( 1-\frac{3}{2}\frac{\vert t\vert }{r_Y}+\frac{1}{2}\frac{\vert t\vert ^3}{r_Y^3}\right)$	$\displaystyle \sigma _Y^2\left[ 1-\frac{s}{\sqrt{2\pi }r_Y}\left( 3-\frac{2s^2}{r_Y^2}\right) \right]$
	if $\vert t \vert \leqslant r_Y$ (0 otherwise)	

### Role of the parameters of the covariance models for the directing function and the coding process

In this section, we investigate the influences of the ranges and sills (variances) of the directing function *T* and the coding process *Y* onto the mean covariance function $\overline{C}_{Z_{s}}$ for a single realization of *Z*.

From Eq. (19), $\overline{C}_{Z_{s}}$ vanishes (or tends to zero) when $C_T$ does, therefore the mean range of single realization of *Z* is equal to the range of *T*, $\overline{r}_{Z_{s}} = r_T$. In particular, a realization *Z* of the SRF displays the same anisotropies as in the latent field *T*.

The covariance function $C_Y$ with range $r_Y$ and sill $\sigma _Y^2=C_Y(0)$ can be written as

20 $$\begin{aligned} C_Y(t) = \sigma _Y^2 \cdot C_{Y_{b}}(t/r_Y), \end{aligned}$$

where $C_{Y_{b}}$ is the covariance function (of same type as $C_Y$) with range and sill equal to 1. Using the definition of $g_Y$ and the change of integration variable $t = r_Y\cdot u$, it follows that

21 $$\begin{aligned}&g_Y(s^2) = {\mathbb {E}}_{t\sim {\mathcal {N}}(0, s^2)}\left[ C_Y(t)\right] = \frac{1}{\sqrt{2\pi }s}\int _{-\infty }^{+\infty }C_Y(t)\exp \left( -\frac{t^2}{2s^2}\right) dt \nonumber \\&\ \ = \frac{\sigma _Y^2}{\sqrt{2\pi }s/r_Y}\int _{-\infty }^{+\infty }C_{Y_{b}}(u)\exp \left( -\frac{t^2}{2s^2/r_Y^2}\right) du = \sigma _Y^2 \cdot g_{Y_{b}}(s^2/r_Y^2). \end{aligned}$$

The mean sill of a single realization of *Z* is then equal to

22 $$\begin{aligned} \overline{\sigma }_{Z_{s}}^2 = \overline{C}_{Z_{s}}(0) = g_Y(0) - g_Y(2\sigma _T^2) = \sigma _Y^2 \left( 1 - g_{Y_{b}}(2\sigma _T^2/r_Y^2) \right) . \end{aligned}$$

Thus, the sill of *Y*, $\sigma _Y^2$, and the ratio $\sigma _T^2/r_Y^2$ controls the mean sill of a single realization of *Z*:

23 $$\begin{aligned} \begin{array}{lclcl} \sigma _T^2/r_Y^2\nearrow \infty &{} \Longrightarrow &{} g_{Y_{b}}(2\sigma _T^2/r_Y^2)\searrow 0 &{} \Longrightarrow &{} \overline{\sigma }_{Z_{s}}^2\nearrow \sigma _Y^2,\\ \sigma _T^2/r_Y^2\searrow 0 &{} \Longrightarrow &{} g_{Y_{b}}(2\sigma _T^2/r_Y^2)\nearrow g_{Y_{b}}(0)=1 &{} \Longrightarrow &{} \overline{\sigma }_{Z_{s}}^2\searrow 0. \end{array} \end{aligned}$$

This means that taking a very small range $r_Y$ compared to $\sigma _T$ vanishes the spatial correlations on *Y* which tends to be a purely white Gaussian noise, and the variance of the resulting field *Z* will be equal to $\sigma _Y^2$. On the opposite, a very large range for *Y* compared to $\sigma _T$ implies almost no variation in the resulting field *Z* (sill decreases to zero), because the coding process *Y*(*t*) will return nearly constant values for the simulated values *t* of the latent field. In particular, the mean sill of a single realization of *Z*, $\overline{\sigma }_{Z_{s}}^2$, does not exceed the sill of *Y*.

**Fig. 1:**
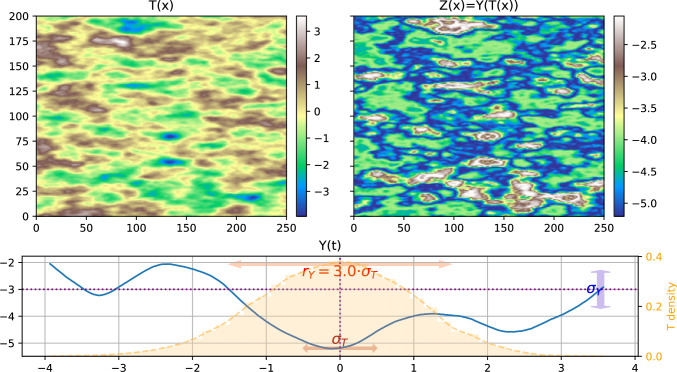
Example of one realization of a substitution random field (SRF). Top left) one realization of the 2D directing function *T*(*x*), bottom) one realization of the 1D coding process *Y*(*t*) (blue line), top right) resulting SRF field $Z(x) = Y(T(x))$. The bottom plot shows additional information: the density distribution of simulated *T* values (in orange), $\sigma _T$, $\sigma _Y$, and $r_Y$ (double arrows), and the mean values of *T* and *Y* as dotted purple lines (respectively vertical and horizontal)

**Fig. 2:**
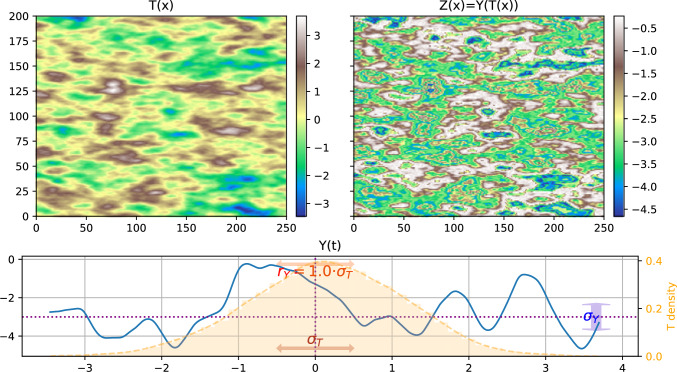
Example of one realization of SRF as in Fig. 1 but with a smaller range for *Y*; top left) field *T*(*x*), bottom) process *Y*(*t*) (blue line), top right) resulting SRF field $Z(x) = Y(T(x))$

To summarize: the range(s) $r_T$ controls the size and shape (anisotropy) of the main structures in single realizations *Z*, the ratio $\sigma _T^2/r_Y^2$ controls the size of the small scale fluctuations within these main structures, and the sill $\sigma _Y^2$ controls the overall amplitude of the simulated values in *Z* (see Figs. 1, 2 in Sect. 5).

## Adding control points on the coding process

As a consequence of the non-ergodicity of the SRF $Z=Y\circ T$, the properties of a single realization of *Z* may significantly differ from one realization to the other. First, as the values of the latent field *T* follow a normal distribution around its mean $\mu _T$ (for instance more than $95\%$ of the *T* values are in the interval $\mu _T\pm 2\cdot \sigma _T$), and providing that the ratio $\sigma _T^2/r_Y^2$ is not too high (i.e. the values do not vary too rapidly through *Y*), the distribution of the simulated *Z* values in one realization strongly depends on the value of $Y(\mu _T)$: a value smaller than $\mu _Y$ (mean value of the Gaussian process *Y*) will favour low values (compared to $\mu _Y$) in the field *Z*, whereas a value greater than $\mu _Y$ will favour high values.

Secondly, it is well known that, whatever the stationary covariance model for the directing function *T*, one can observe (in more than one dimension) that the region with values close to $\mu _T$ in the latent field, $\{x\in \Omega \,\ T(x) \approx \mu _T\}$, is well connected, whereas the low-value and high-value regions are made up of several isolated zones (Zinn and Harvey 2003). Hence, in the field $Z(x) = Y(T(x))$ the region with values close to $Y(\mu _T)$ will be well connected (see Figs. 1, 2 in Sect. 5).

According to this finding, a simple idea consists of imposing the value of *Y* at $\mu _T$ to control what is connected in the realizations of *Z*. Then, we use a *control point* $Y(\mu _T)=y_{\mu _T}$ and consider the conditional simulation

24 $$\begin{aligned} Z \,\vert \, Y(\mu _T)=y_{\mu _T}, \end{aligned}$$

the constraint on *Y* (control point) indicating the region around the specified value $y_{\mu _T}$ that has to be connected. Changing this value allows for exploring several scenarios of connectivity patterns. Note that in general an ensemble of control points on the coding process, $\{Y(t_k)=y_k\}_{k\in K}$, can be considered.

### Ensemble distribution of SRF conditioned to $Y(\mu _{T})=y_{\mu _{T}}$

The process *Y* is multi-Gaussian with a covariance function $C_Y$, a mean $\mu _Y$ (and a variance $\sigma _Y^2=C_Y(0)$), then $Y(\mu _T+t)$ given $Y(\mu _T)=y_{\mu _T}$ follows the normal distribution

25 $$\begin{aligned}{} & {} \left( Y(\mu _T + t)\,\vert \, Y(\mu _T)=y_{\mu _T}\right) \sim {\mathcal {N}}\left( \mu _Y + \frac{C_Y(t)}{\sigma _Y^2}(y_{\mu _T}-\mu _Y),\right. \nonumber \\{} & {} \quad \left. \sigma _Y^2-\frac{C_Y(t)^2}{\sigma _Y^2}\right) . \end{aligned}$$

Hence, the distribution of $Z(x) = Y(T(x))$ given $Y(\mu _T)=y_{\mu _T}$ can be derived by computing its CDF

26 $$\begin{aligned}&F_{Z\, \vert \, Y(\mu _T)=y_{\mu _T}}(z) = {\mathbb {P}}\left( Z(x) \leqslant z\,\vert \, Y(\mu _T)=y_{\mu _T}\right) \nonumber \\&= \sum _{t} {\mathbb {P}}\left( Y(t)\leqslant z, T(x)=t \,\vert \, Y(\mu _T)=y_{\mu _T}\right) \nonumber \\&= \sum _{t} {\mathbb {P}}\left( Y(t)\leqslant z\,\vert \, Y(\mu _T)=y_{\mu _T}\right) \cdot {\mathbb {P}}(T(x)=t) \nonumber \\&= \sum _{t} {\mathbb {P}}\left( Y(\mu _T+t)\leqslant z\,\vert \, Y(\mu _T)=y_{\mu _T}\right) \cdot {\mathbb {P}}(T(x)-\mu _T=t) \nonumber \\&= {\mathbb {E}}_{t\sim {\mathcal {N}}(0, \sigma _T^2)}\Phi \left( \frac{z-\left( \mu _Y + \frac{C_Y(t)}{\sigma _Y^2}(y_{\mu _T}-\mu _Y)\right) }{\left( \sigma _Y^2-\frac{C_Y(t)^2}{\sigma _Y^2}\right) ^{1/2}}\right) \end{aligned}$$

where the third equality holds because of the independence of *T* and *Y*, and the last equality follows from the conditional CDF of the distribution in Eq. (25) expressed with the CDF $\Phi$ of ${\mathcal {N}}(0, 1)$ and from $T(x)\sim {\mathcal {N}}(\mu _T, \sigma _T^2)$.

Note that the CDF in Eq. (26) is an *ensemble distribution*, which can be estimated from an ensemble of realizations $Z_i\,\vert \, Y(\mu _T)=y_{\mu _T}$, as in Eq. (15),

27 $$\begin{aligned} F_{Z\, \vert \, Y(\mu _T)=y_{\mu _T}}(z) \approx {\mathbb {E}}_{i\in I}\left[ F_{Z_i\, \vert \, Y(\mu _T)=y_{\mu _T}}(z)\right] . \end{aligned}$$

### Specifying a target distribution

The goal is to obtain realizations of the SRF with values following a marginal cumulative density function (CDF) *G*.

Knowing the ensemble distribution $F_Z$, it is possible to transform the values of *Z* by applying the anamorphosis $H=G^{-1} \circ F_{Z}$, then $F_{H\circ Z} = F_Z\circ H^{-1} = G$ as wanted. Thus, for any realization, the transformation is $Z_i(x)\mapsto H\circ Z_i(x) = G^{-1}(F_Z(Z_i(x))$.

For unconstrained SRF simulation, $F_Z$ is the CDF of ${\mathcal {N}}(\mu _Y, \sigma _Y^2)$ (see Eq. (2)), whereas for SRF controlled by the value of *Y* at the mean of *T*, i.e. $Z \,\vert \, Y(\mu _T)=y_{\mu _T}$, the CDF given in Eq. (26) can be used to define the anamorphosis $H=G^{-1}\circ F_{Z\, \vert \, Y(\mu _T)=y_{\mu _T}}$.

The ensemble distribution can be expressed as the mean distribution of all the single realizations (see Eqs. (15) and (27)). Hence, an idea to reduce the spread of the ensemble of the single CDFs is to apply the anamorphosis $H_i=G^{-1}\circ F_{Z\, \vert \, Y(\mu _T)=Y_i({\text {mean}}(T_i))}$ to the *i*-th realization $Z_i = Y_i\circ T_i$. In this way, each realization is transformed using its own anamorphosis that accounts for the value of the underlying realization of the coding process at the mean of the simulated *T* field. Note that one single realization could be transformed by an anamorphosis based on its empirical CDF itself, however, this latter often displays sharp transitions and should then be smoothed to get a reliable anamorphosis function.

## Conditional SRF simulations with control points on the coding process

Conditioning SRFs can be done using a Gibbs sampler (Lantuéjoul 2002; Emery 2008). In this section, we show that this strategy can still be used in the presence of control points in the coding process. Let $\{Y(t_k)=y_k\}_{k\in K}$ a set of control points on *Y* and consider a set of conditioning data $\{Z(x_j)=z_j\}_{j\in J}$. The aim is then to generate conditional simulations of

28 $$\begin{aligned} Z \,\vert \, \{Z(x_j)=z_j\}_{j\in J}, \{Y(t_k)=y_k\}_{k\in K}. \end{aligned}$$

The following algorithm allows generating one conditional realization on a domain $\Omega$.

1. Generate $\{t_j=T(x_j)\}_{j\in J}$ conditional to $\{Z(x_j)=z_j\}_{j\in J}$ and $\{Y(t_k)=y_k\}_{k\in K}$.
2. Generate a realization of *T* on $\Omega$ conditional to $\{t_j=T(x_j)\}_{j\in J}$.
3. Generate a realization of *Y* (on a set containing $T(\Omega )$) conditional to $\{Y(t_j)=z_j\}_{j\in J}$ and $\{Y(t_k)=y_k\}_{k\in K}$.
4. Retrieve $$Z(x) = Y(T(x))$$, $$x\in \Omega$$.

Whereas the steps (2) and (3) consist of classical conditional multi-Gaussian simulations, the step (1) is more difficult: the aim is to generate values $t_j$ that are the outputs of *T* at the conditioning locations $x_j$, and the inputs of *Y* sent to the conditioning values $z_j$. Hence, these values $t_j$ must be consistent with the covariance of *T* regarding the locations $x_j$ and with the covariance of *Y* regarding the locations $t_k$ and the values $z_j$ and $y_k$. This step (1) is done with a Gibbs sampler as follows.

- Initialization: generate $\{t_j=T(x_j)\}_{j\in J}$, unconditional simulation of *T* at the conditioning locations $x_j$.
- Choose randomly (and uniformly) one index $j_0 \in J$.
- Generate a candidate value $t'_{j_0} = T(x_{j_0})\,\vert \,\{t_j=T(x_j)\}_{j\in J, j\ne j_0}$.
- Compute the Metropolis ratio (see appendix A)
  29 $$\begin{aligned} r_{j_0} = \frac{{\mathbb {P}}\left( Y(t'_{j_0}) = z_{j_0}\,\vert \, \{Y(t_j)=z_j\}_{j\in J, j\ne j_0}, \{Y(t_k)=y_k\}_{k\in K}\right) }{{\mathbb {P}}\left( Y(t_{j_0}) = z_{j_0}\,\vert \, \{Y(t_j)=z_j\}_{j\in J, j\ne j_0}, \{Y(t_k)=y_k\}_{k\in K}\right) }, \end{aligned}$$
  and update $t_{j_0}$: accept the candidate $t'_{j_0}$ with probability $p_{j_0} = \min (1, r_{j_0})$, i.e. draw *u* uniformly in [0, 1], and set $t_{j_0}=t'_{j_0}$ if $u \leqslant p_{j_0}$, and let $t_{j_0}$ unchanged otherwise.
- Go to step (1b) until a given number of iterations is reached.

This algorithm produces a Markov chain $\left( \left\{ t_j^{(n)}\right\} _{j\in J}\right) _{n\geqslant 1}$ following the distribution

30 $$\begin{aligned}{} & {} \pi \left( \{t_j\}_{j\in J}\right) ={\mathbb {P}}\left( \{t_j=T(x_j)\}_{j\in J}\,\vert \, \{Z(x_j)=z_j\}_{j\in J}, \right. \nonumber \\{} & {} \quad \left. \{Y(t_k)=y_k\}_{k\in K}\right) \end{aligned}$$

as wanted in step (1). Note that the acceptation probability $p_{j_0}$ in step (1d) can be defined more generally as $p_{j_0} = f(r_{j_0})$, where *f* is a function defined on positive real number with values in ]0, 1] verifying $f(u)=u\cdot f(1/u)$. The function $\min (1, u)$ is such a function, $u/(1+u)$ another one. Note finally that as for steps (2) and (3), step (1c) and the computation of the Metropolis ratio (Eq. (29)) in step (1d) involve only classical conditional multi-Gaussian simulations. The numerator (as well as the denominator) is treated by solving a kriging system to retrieve the mean and variance of the corresponding Gaussian distribution (the density function is used instead of $\mathbb {P(.)}$).

Note that, provided that the ensemble distribution of the SRF is known *a priori* (before generating realizations), the anamorphosis *H* discussed in Sect. 3.2 could be used to approximately fit a target distribution. In this situation, the data values $z_j=Z(x_j)$ are first transformed via $H^{-1}$, i.e. ${\tilde{z}}_j = H^{-1}(z_j)$, then the conditional SRF simulation $\widetilde{Z}$ is done (given $\widetilde{Z}(x_j) = {\tilde{z}}_j$), and finally the resulting field is back-transformed via *H*, i.e. $Z(x)= H\left( \widetilde{Z}(x)\right)$. However, as a conditioning data point may influence any point in the simulation grid (non-ergodicity of *Z*), the ensemble distribution of SRF is no longer the same one as for unconditional simulation; therefore, the anamorphosis only helps guide the realizations towards the target distribution, but the final ensemble CDF will not fit it exactly.

## Illustrations

In the following examples, Matérn covariances are used for the directing function and the coding process. The Matérn covariance model (Stein 1999) of parameter $\nu$ is given by the function (in one dimension) defined as

31 $$\begin{aligned} M_{\nu }(h) = \sigma ^2 \cdot \frac{1}{2^{\nu -1}\Gamma (\nu )}\left( \sqrt{2\nu }\frac{\vert h\vert }{r}\right) ^{\nu }K_\nu \left( \sqrt{2\nu }\frac{\vert h\vert }{r}\right) , \end{aligned}$$

where $K_\nu$ is the modified Bessel function of the second kind of order $\nu$ (Olver et al. 2010). If $h \rightarrow 0$, then $M_{\nu }(h)\rightarrow \sigma ^2$, the variance of the model. The parameter *r* is a scale parameter linked to the effective range, $r_{\textit{eff}}\,$, such that $M_{\nu }(h) < 0.05\cdot \sigma ^2$ for $h>r_{\textit{eff}}\,$; given $\nu$, one can numerically compute *r* as a function of $r_\textit{eff}$ and inversely. The advantage of such a model is that the parameter $\nu$ controls the smoothness of the resulting random fields: for $\nu = 1/2$, one gets the exponential model of effective range 3*r*, $M_{1/2}(h) = \sigma ^2 \exp \left( -\frac{\vert t\vert }{r}\right)$, and if $\nu \rightarrow +\infty$, then $M_{\nu }(h)\rightarrow \sigma ^2 \exp \left( -\frac{t^2}{2r^2}\right)$, the Gaussian model of effective range $\sqrt{6}r$ (see expression of classical model in Table 1).

### Simple case and influence of the range of the coding process

Two-dimensional SRFs $Z=Y(T(x))$ are generated in a simulation domain $\Omega$ of $250 \times 200$ cells. For the latent field *T*, a Matérn covariance model of parameter $\nu _T=3/2$ is used, with effective ranges of 45 and 15 (in number of cells) along horizontal and vertical axis respectively. The variance is set to $\sigma _T^2= 1$ and the mean to $\mu _T=0$. Note that for convenience these values for the variance and the mean for *T* can be kept constant because the final range of values in *Z* is controlled by the parameters of the coding process *Y*.

For the following examples, the mean of *Y* is set arbitrarily to $\mu _Y=-3$, its variance to $\sigma _Y^2= 2$, and a (uni-dimensional) Matérn covariance model of parameter $\nu _Y=3$ is chosen (rather smooth). Different values of the effective range $r_Y$ are used in the following examples, they are taken as a given coefficient times $\sigma _T$ (according to the discussion in Sect. 2.4).

Figure 1 shows one example of a realization of the SRF *Z*(*x*) with $r_Y = 3\cdot \sigma _T$. The figure shows the simulation used for the directing function *T*(*x*) and the coding process *Y*(*t*). The most important feature is that the intermediate values (around 0) are connected over large distances in the simulation of *T*(*x*) while the low values (around -5) are those which are connected in *Z*(*x*). Depending on the simulation of the coding process, the range of connected values will change. This feature is crucial since it will allow covering a broader range of connectivity patterns than the GRFs. Figure 2 shows another example with a smaller correlation length for the coding process, $r_Y = 1\cdot \sigma _T$. When comparing Figs. 1 and 2, we see that the sizes of the main structures in the fields *T* and *Z* are similar in both figures, but there are more inner variations within the large structures when $r_Y$ is smaller, as expected. We also observe that large values in the *Z* field in Fig. 2 are more frequent and more connected than in Fig. 1. This is not related to the parameter $r_Y$, but it is explained by the fact that the value of $Y(\mu _T)$ is low in Fig. 1 (resp. high in Fig. 2) compared to $\mu _Y$ (see the dotted lines in the figures), as discussed in Sect. 3.

### Ensemble of unconstrained SRF simulations

Distributions and covariances computed from an ensemble of SRF realizations are illustrated in this section. The same simulation domain and the same parameters as in the previous section are considered for both the directing function *T* and the coding process *Y*, except the range for *Y* set to $r_Y = 2\sigma _T$.

**Fig. 3:**
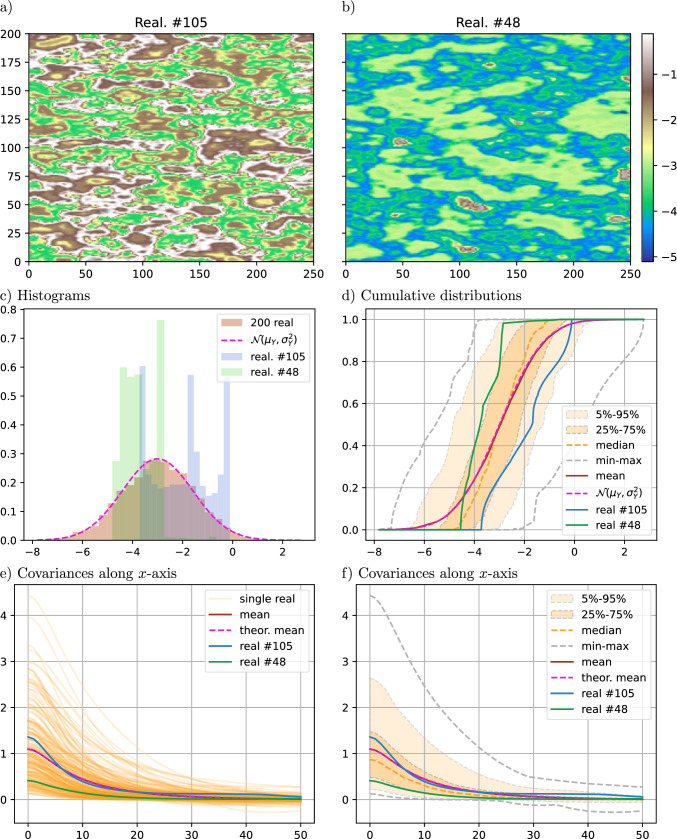
Results for an ensemble of 200 realizations of unconstrained SRF; a–b) two selected realizations (same color bar), c–f) statistics computed from the ensemble and theoretical result (pink)

An ensemble of 200 realizations of $Z(x)=Y(T(x))$ is generated. Figure 3 shows two realizations from this ensemble as well as their density distributions and covariances. For each realization $Z_i$, the empirical cumulative distribution function $F_{Z_i}$ and the covariance function along *x*-axis, $C_{Z_i}(h) = {\text {Cov}}_{x\in \{x\in \Omega :x+h\in \Omega \}}\left( Z_i(x), Z_i(x+h)\right)$ with horizontal lag vector *h*, are computed. Statistics (min, max, mean, and quantiles) are then retrieved from these curves. For the CDF (Fig. 3d), the mean curve ${\mathbb {E}}_{i}\left[ F_{Z_i}\right]$ (in brown in the figure) is similar to the theoretical CDF of ${\mathcal {N}}(\mu _Y, \sigma _Y^2)$ (in pink), according to Eq. (15). For the covariance (Fig. 3e, f), the mean curve ${\mathbb {E}}_{i}\left[ C_{Z_i}(h)\right]$ (in brown) also fits well the theoretical function (in pink) given by Eq. (19) (with $g_Y$ computed empirically). The two realizations displayed in Fig. 3a, b are selected such that their empirical CDFs are respectively below and above the quantiles $25\%$ and $75\%$ of the theoretical CDF at $\mu _Y$. This figure shows the wide variability of the marginal distributions and covariances of the realizations obtained with the SRF model. In the following sections, we will use a control point on *Y* and histogram transforms to better constrain the realizations.

### SRF simulations with a control point

**Fig. 4:**
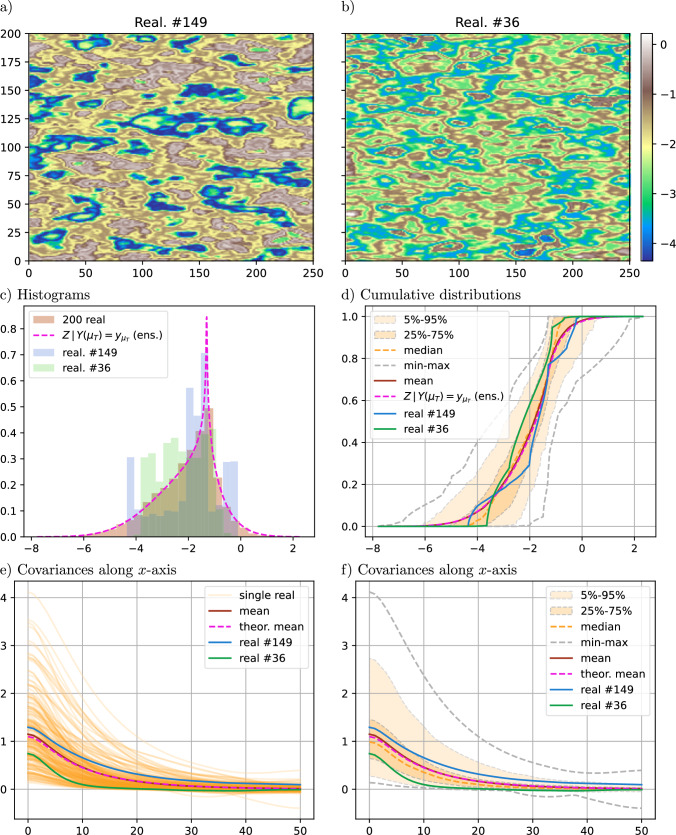
Results for an ensemble of 200 realizations of SRF $Z(x)\,\vert \, Y(\mu _T)=y_{\mu _T}$, with $y_{\mu _T} = \mu _Y + 1.2 \sigma _Y$; a-b) two selected realizations (same color bar), c-f) statistics computed from the ensemble and theoretical result (pink)

The same set-up as in the previous section is considered but a control point is added to guide the simulations. An ensemble of 200 realizations of the constrained SRF $Z(x)\,\vert \, Y(\mu _T)=y_{\mu _T}$ is generated. The value of *Y* at the mean of *T* is set to $y_{\mu _T}=\mu _Y + 1.2 \sigma _Y$. The results are shown in Fig. 4.

Figure 4d shows that the mean CDF curve ${\mathbb {E}}_{i}\left[ F_{Z_i\, \vert \, Y(\mu _T)=y_{\mu _T}}\right]$ (in brown in the figure) is very close to the theoretical CDF of $F_{Z\, \vert \, Y(\mu _T)=y_{\mu _T}}$ (in pink) given by Eq. (26) (which is no longer Gaussian). Figure 4e, f show that the mean covariance curve ${\mathbb {E}}_{i}\left[ C_{Z_i\, \vert \, Y(\mu _T)=y_{\mu _T}}(h)\right]$ (in brown) does not deviate much from the theoretical covariance function given by Eq. (19) (not accounting for the control point). The two realizations displayed in Fig. 4a, b are selected such that their empirical CDFs are respectively below and above the quantiles $25\%$ and $75\%$ of the theoretical CDF at $1/2(\mu _Y+y_{\mu _T})$. Note that the high-value region is rather well connected in both these realizations, but their covariance and distribution are rather different.

Compared to the unconstrained SRF simulations (Sect. 5.2), the distributions of the simulated values in every realization are less spread around the theoretical mean distribution (compare Figs. 3c, d and  4c, d), whereas the covariance curves show similar variability (compare Figs. 3e, f and  4e, f).

### Conditional SRF

**Fig. 5:**
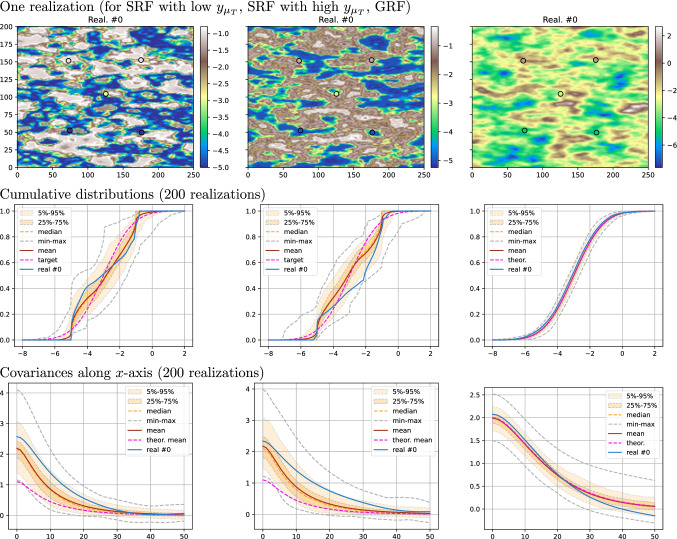
Results of conditional simulations: left column) SRF with low $y_{\mu _T}$, case (1); middle column) SRF with high $y_{\mu _T}$, case (2); right column) GRF *X*, case (3). Top row) first realization; middle row) cumulative distribution; bottom row) covariance along *x*-axis. Statistics are computed over 200 realizations. See text for details

This section compares conditional simulations obtained with the SRF (*Z*) and GRF (*X*) models. Five conditioning data points are considered in the simulation grid (same domain as in the previous examples). The data values are respectively set to $-5$ and $-1$ for the two points in the lower and upper parts of the simulation grid, and to $-3$ for the point near the center (see the circles in the first row of Fig. 5). For the SRF, we use the same parameters as those employed in Sect. 4, with the target distribution ${\mathcal {N}}(\mu _Y, \sigma _Y^2) = {\mathcal {N}}(-3, 2)$, that is the anamorphosis $H=G^{-1}\circ F_{Z\, \vert \, Y(\mu _T)=y_{\mu _T}}$ is used, where *G* is the CDF of the target distribution. As mentioned in the last paragraph of Sect. 4, the target will not be fitted exactly. For the GRF, we propose to use the covariance model used for the latent field *T*, but with the variance and mean of *Y* to fit the target distribution. The following three cases are considered.

1. SRF with a low value as control point, $y_{\mu _T} = \mu _Y - 1.2\cdot \sigma _Y$, and anamorphosis.
2. SRF with a high value as control point, $y_{\mu _T} = \mu _Y + 1.2\cdot \sigma _Y$, and anamorphosis.
3. GRF *X* based on the same covariance model as *T*, except the sill set to $\sigma _X^2 = \sigma _Y^2 =2$, and the mean set to $\mu _X = \mu _Y=-3$. (No anamorphosis.)

**Fig. 6:**
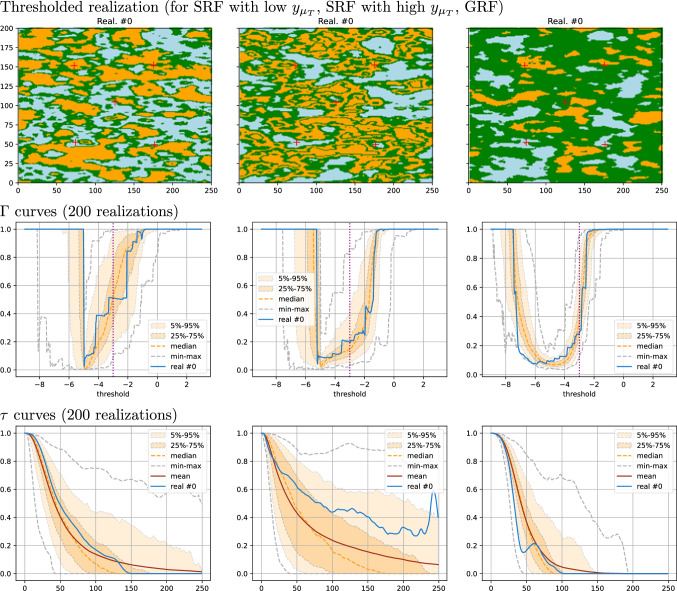
Results of conditional simulations: left column) SRF with low $y_{\mu _T}$, case (1); middle column) SRF with high $y_{\mu _T}$, case (2); right column) GRF *X*, case (3). Top row) first realization thresholded; middle row) $\Gamma (v)$ curves; bottom row) $\tau (h)$ curves along *x*-axis for high-value region in thresholded realizations. Statistics are computed over 200 realizations. See text for details

In the three cases, 200 conditional realizations are generated. The results are shown in Figs. 5 and 6.

In Fig. 5, the first realization is displayed for each case in the top row, and the statistics on the cumulative distribution and the covariance along the *x*-axis of every realization in the middle and bottom rows respectively. For the SRF, the distributions are guided by the target one, but the tails do not match well the target, whereas, for the GRF the distribution matches very well the target distribution everywhere. The theoretical mean covariance, computed without accounting for the control point, the conditioning data, and the anamorphosis, is shown as a pink dashed line on the two first plots. The presence of conditioning data and the anamorphosis explain the deviation from the actual mean of the covariances of the single realizations. Note that the covariance of the GRF is defined as $\sigma _Y^2$ times the covariance of the predicting function *T*, $C_X(h) = \sigma _Y^2 \cdot C_T(h)$, thus the last row in Fig. 5 shows how the coding process *Y* transforms the covariance of *T* into the covariance of *Z*.

We then consider the connectivity of the simulated fields. The top row of Fig. 6 shows the first realization of each case, thresholded in three categories: in blue (resp. orange) the cells having a value less than $\mu _Y-\sigma _Y$ (resp. greater than $\mu _Y+\sigma _Y$) and in green the remaining cells (with values between $\mu _Y\pm \sigma _Y$). The realization of the SRF for case (1) has the low values (blue) well connected, whereas for case (2) the high values (orange) are well connected. For the GRF, the middle values (green) are well connected. This visual analysis confirms that the SRF simulations seem to have a different type of connectivity than the GRF simulations.

To quantify the connectivity properties, we use two metrics: *the* $\Gamma$ *connectivity function* $\Gamma (v)$ and *the connectivity function* $\tau (h)$. These metrics are described in detail in Renard and Allard (2013). They are defined for any continuous field *Z* on a grid $\Omega$ as follows. For a value *v*, the subset of $\Omega$ composed of the cells where *Z* is less than or equal to *v*, $S_v=\{x\in \Omega :\, Z(x)\leqslant v\}$, is considered, and the number *N*(*v*) of its connected components, and their respective number of cells, $n_1, \ldots , n_{N(v)}$, are retrieved. Then, $\Gamma (v)$ is defined by Renard and Allard (2013) as the probability that two cells randomly chosen in *S*(*v*) are connected (i.e. belong to the same connected component). It can be expressed as

32 $$\begin{aligned} \Gamma (v) = \frac{1}{\vert S(v)\vert ^2}\sum _{i=1}^{N(v)} n_i^2, \end{aligned}$$

where $\vert S(v)\vert = \sum _{i=1}^{N(v)} n_i$ is the total number of cells in *S*(*v*). Note that $\Gamma (v)$ is set to 1 if *S*(*v*) is empty. When this probability is equal to 1, all the grid cells having a simulated value lower than *v* belong to the same connected component. When the probability is close to zero, the set of cells with *Z* lower than *v* is highly fragmented and composed of many unconnected subsets. For each realization, the curve $\Gamma (v)$ is computed and shown in the middle row of Fig. 6. A complete characterization of the connectivity pattern would require in addition the computation and analysis of the $\Gamma _c(v)$ function for the complementary set corresponding to the values higher than the threshold. But, this analysis would go beyond the scope of this paper. Here, we can already observe and conclude from the middle row of Fig. 6 that the three $\Gamma (v)$ functions are very different. On those plots (middle row of Fig. 6), the vertical dotted line indicates the abscissa value $\mu _Y$. For the GRF, the $\Gamma$ curve rapidly increases around this value, whereas for the SRF in case (1) it starts to increase before, reflecting the good connection of low values. In case (2), the curve remains longer with low probabilities, meaning that the connections of the values below the threshold are broken by the connections of the high-value areas.

The connectivity function $\tau (h)$ is another tool to quantify the connectivity. It provides more information (about the size of the connected components) but is restricted to indicator (categorical) random functions. Here we apply it only for *the high values* of *Z*. For a realization *Z*, the set $M = \Omega {\setminus } S_{\mu _Y+\sigma _Y}$ is considered, *i.e.* $x\in M \iff Z(x) > \mu _Y+\sigma _Y$, which corresponds to the orange region displayed in the top row of Fig. 6 for the first realization. Then, the probability that two grid cells *x* and $x+h$ in *M* distant from a horizontal lag vector *h* are in the same connected component, is computed and written

33 $$\begin{aligned} \tau (h) = {\mathbb{P}}(x\leftrightarrow x+h\,\vert \, x, x+h\in M), \end{aligned}$$

the two-head arrow meaning that a path of adjacent cells within the set *M* and linking the two cells exists. Statistics on the $\tau$ curves computed for each realization in each case are shown in the bottom row of Fig. 6. One observes that the SRF with the high control point value (case (2), middle column) shows higher probabilities of connection when *h* increases. This means that the probability of observing larger connected components is higher in this case. Although the range of the covariance is longer for the GRF than for the SRF, the $\tau$ curves decrease faster (right column) and therefore the probability of getting large connected components drops rapidly to zero in that situation. This confirms that the SRF model can cover a much larger range of patterns for the connected components than the GRF model.

## Conclusions

Stationary multi-Gaussian random fields (GRF) are parameterized by their mean and covariance model. They are easy to define and simulate but their connectivity patterns cannot be controlled. In two or three-dimensional simulations, low and high-value regions always form isolated zones, whereas the middle-value (near to mean) region is well connected. Using multi-Gaussian fields may therefore lead to an underestimation of the uncertainty when predicting flow and transport (Gómez-Hernández and Wen 1998; Zinn and Harvey 2003; Kerrou et al. 2008) because simulations with highly connected or highly disconnected hydraulic conductivity values would not be simulated by this technique. This paper investigated therefore the feasibility of using substitution random field (SRF) to generate fields having a broader (and if possible controlled) distribution of connectivity patterns.

To investigate this question, we used substitution random fields built by composing two stationary GRFs, the directing function *T*, and the coding process *Y* to get $Z=Y\circ T$. This technique is parsimonious because it uses the simple parameterization of GRFs with their mean and covariance function, but it allows enriching the generated spatial features, in particular in terms of connectivity.

Assuming that the directing function *T* is second-order stationary with bounded variogram (finite variance), we have shown that the resulting SRF *Z* is not ergodic in the mean and the covariance. It means that the statistical properties of *Z* cannot be derived from a single realization or field observations. Nevertheless, we have established an analytical expression for the mean of the covariance function describing a large ensemble of realizations. Furthermore, adding a control point on the coding process *Y*, consisting of imposing the value of *Y* at the mean of *T*, allows to control partly the connectivity structures of the simulated fields: the region with values close to the prescribed value at the control point tends to be well connected. Moreover, the mean distribution over the ensemble of realizations can be expressed with respect to this control point. We show how the simulation of this type of SRF can handle conditioning data with a Gibbs sampler. Thus, we provide an algorithm able to generate conditional simulation with partial control of the connectivity patterns.

However, the type of SRF tested in this paper suffers from several drawbacks. First, the target distribution is only approximately reproduced for each single realization, especially around the extreme values. Indeed, for instance, fields with the high-value region well connected tend to present a peak for the high values in the histogram. This peak is difficult to transform in a long tail as in a Gaussian distribution. The underlying reason for this phenomenon is that it is not possible to create connected paths over a long distance (an infinite cluster) in a random field if the proportion of cells involved in this path is too small. Secondly, identifying the parameters of the underlying covariance models is difficult. The range of the coding process should be set relative to the standard deviation of the directing function. But more generally, the non-ergodicity of this model makes the inference of the parameters difficult. This suggests that further research should be conducted in this field before the method can be applied easily for field applications.

In summary, although the proposed method is still a bit difficult to constrain because the statistics and the connectivity patterns vary strongly between the realizations, this can also be seen as an advantage because it allows mitigating the risk of underestimation of uncertainty. This may be very important for groundwater flow and solute transport or other applications deeply impacted by connectivity structures.

## Appendix A Metropolis ratio in the Gibbs sampler

The Metropolis ratio (Eq. (29)) in the Gibbs sampler algorithm of Sect. 4 is obtained as follows (adapted from Lantuéjoul (2002)). Let $\pi$ be the distribution over $\{t_j\}_{j\in J}$ that has to be sampled in step (1) for conditional SRF given by Eq. (28), i.e.

A1 $$\begin{aligned} \pi (t_J) = {\mathbb {P}}\left( T(x_J)=t_J\,\vert \,Z(x_J)=z_J, Y(t_K)=y_K \right) \end{aligned}$$

where $t_J = \{t_j\}_{j\in J}$, and similarly for $x_J$, $z_J$, and $t_K$, $y_K$.

For a given index $j_0$, let $J_0 = J\setminus \{j_0\}$ be the ensemble of indices in *J* private of the index $j_0$, and for a candidate value $t'_{j_0}$, let $t'_J$ be the vector with the value $t'_{j_0}$ at index $j_0$ and with $t'_{J_0} = t_{J_0}$, i.e. the vector obtained from $t_J$ by updating only the $j_0$-th component. Hence, the proposal distribution corresponding to steps (1b-1c) (Sect. 4) in the Gibbs sampler algorithm, from $t_J$ to the candidate $t'_J$ is expressed as

A2 $$\begin{aligned} Q(t_J, t'_J) = \frac{1}{\vert J\vert }{\mathbb {P}}\left( T(x_{j_0})=t'_{j_0}\,\vert \, T(x_{J_0})=t_{J_0}\right) . \end{aligned}$$

The Metropolis ratio to get a chain whose distribution converges towards $\pi$ (invariant distribution) is then given by (Robert and Casella 2004)

A3 $$\begin{aligned} r(t_J, t'_J) = \frac{\pi (t'_J)\cdot Q(t'_J, t_J)}{\pi (t_J)\cdot Q(t_J, t'_J)}. \end{aligned}$$

Developing the expression in Eq. (A1), we have

A4 $$\begin{aligned} \pi (t_J)&= {\mathbb {P}}\left( T(x_{j_0})=t_{j_0}, T(x_{J_0})=t_{J_0}\,\vert \, Z(x_J)=z_J, Y(t_K)=y_K\right) \nonumber \\&= {\mathbb {P}}\left( T(x_{J_0})=t_{J_0}\,\vert \, Z(x_J)=z_J, Y(t_K)=y_K\right) \cdot \nonumber \\&\quad \ {\mathbb {P}}\left( T(x_{j_0})=t_{j_0}\,\vert \, T(x_{J_0})=t_{J_0}, Z(x_J)=z_J, Y(t_K)=y_K\right) . \end{aligned}$$

The last factor above can be written as

A5 $$\begin{aligned}&{\mathbb {P}}\left( T(x_{j_0})=t_{j_0}\,\vert \, T(x_{J_0})=t_{J_0}, Z(x_J)=z_J, Y(t_K)=y_K\right) \nonumber \\&\quad = {\mathbb {P}}\left( T(x_{j_0})=t_{j_0}\,\vert \, T(x_{J_0})=t_{J_0}, Y(t_{J_0})=z_{J_0}, Z(x_{j_0})=z_{j_0}, Y(t_K)=y_K\right) \nonumber \\&\quad = \frac{{\mathbb {P}}\left( T(x_{j_0})=t_{j_0}, Z(x_{j_0})=z_{j_0}\,\vert \, T(x_{J_0})=t_{J_0}, Y(t_{J_0})=z_{J_0}, Y(t_K)=y_K\right) }{{\mathbb {P}}\left( Z(x_{j_0})=z_{j_0}\,\vert \, T(x_{J_0})=t_{J_0}, Y(t_{J_0})=z_{J_0}, Y(t_K)=y_K\right) }. \end{aligned}$$

By independence of *T* and *Y*, the numerator above can be written as

A6 $$\begin{aligned}&{\mathbb {P}}\left( T(x_{j_0})=t_{j_0}, Z(x_{j_0})=z_{j_0}\,\vert \, T(x_{J_0})=t_{J_0}, Y(t_{J_0})=z_{J_0}, Y(t_K)=y_K\right) \nonumber \\&= {\mathbb {P}}\left( T(x_{j_0})=t_{j_0}, Y(t_{j_0})=z_{j_0}\,\vert \, T(x_{J_0})=t_{J_0}, Y(t_{J_0})=z_{J_0}, Y(t_K)=y_K\right) \nonumber \\&= {\mathbb {P}}\left( T(x_{j_0})=t_{j_0}\,\vert \, T(x_{J_0})=t_{J_0}\right) \cdot {\mathbb {P}}\left( Y(t_{j_0})=z_{j_0}\,\vert \, Y(t_{J_0})=z_{J_0}, Y(t_K)=y_K\right) . \end{aligned}$$

Hence, inserting in Eq. (A3) the expression of the proposal distribution *Q* (Eq. (A2)) and the expression of $\pi$ obtained by gathering Eqs. (A4-A6), we obtain the Metropolis ratio

A7 $$\begin{aligned} r(t_J, t'_J) = \frac{{\mathbb {P}}\left( Y(t'_{j_0})=z_{j_0}\,\vert \, Y(t_{J_0})=z_{J_0}, Y(t_K)=y_K\right) }{{\mathbb {P}}\left( Y(t_{j_0})=z_{j_0}\,\vert \, Y(t_{J_0})=z_{J_0}, Y(t_K)=y_K\right) } \end{aligned}$$

as given by Eq. (29) in step (1d) of the algorithm in Sect. 4.

## References

[CR1] Allard D, Emery X, Lacaux C, Lantuéjoul C (2020). Simulating space-time random fields with nonseparable gneiting-type covariance functions. Stat Comput.

[CR2] Chilès JP, Delfiner P (1999). Geostatistics: modeling spatial uncertainty.

[CR3] Emery X (2008). Substitution random fields with gaussian and gamma distributions: theory and application to a pollution data set. Math Geosci.

[CR4] Gómez-Hernández JJ, Wen X-H (1998). To be or not to be multi-gaussian? A reflection on stochastic hydrogeology. Adv Water Resour.

[CR5] Goodfellow I, Bengio Y, Courville A (2016) Deep learning. MIT Press. http://www.deeplearningbook.org

[CR6] Kerrou J, Renard P, Franssen H-JH, Lunati I (2008). Issues in characterizing heterogeneity and connectivity in non-multiGaussian media. Adv Water Resour.

[CR7] Knudby C, Carrera J (2005). On the relationship between indicators of geostatistical, flow and transport connectivity. Adv Water Resour.

[CR8] Lantuéjoul C (1993). Substitution random functions.

[CR9] Lantuéjoul C (2002). Geostatistical simulation: models and algorithms.

[CR10] Mariethoz G, Caers J (2014). Multiple-point geostatistics: stochastic modeling with training images.

[CR11] Olver FWJ, Lozier DW, Boisvert RF, Clark CW (2010). NIST handbook of mathematical functions.

[CR12] Rasmussen CE, Williams CK (2006). Gaussian processes for machine learning.

[CR13] Renard P, Allard D (2013). Connectivity metrics for subsurface flow and transport. Adv Water Resour.

[CR14] Robert CP, Casella G (2004). Monte Carlo statistical methods.

[CR15] Stein ML (1999). Interpolation of spatial data: some theory for kriging.

[CR16] Tyukhova AR, Willmann M (2016). Connectivity metrics based on the path of smallest resistance. Adv Water Resour.

[CR17] Zinn B, Harvey CF (2003). When good statistical models of aquifer heterogeneity go bad: a comparison of flow, dispersion, and mass transfer in connected and multivariate gaussian hydraulic conductivity fields. Water Resour Res.

